# Development and Validation of a Questionnaire to Measure Knowledge of and Attitude toward COVID-19 among Nursing Students in Greece

**DOI:** 10.3390/nursrep10020012

**Published:** 2020-11-16

**Authors:** Athina E. Patelarou, Theocharis Konstantinidis, Evangelia Kartsoni, Enkeleint A. Mechili, Petros Galanis, Michail Zografakis-Sfakianakis, Evridiki Patelarou

**Affiliations:** 1 Department of Nursing, Faculty of Health Sciences, Hellenic Mediterranean University, Estavromenos, 71140 Heraklion, Greece; harriskon@hmu.gr (T.K.); evikartsoni@gmail.com (E.K.); mzografakis@hmu.gr (M.Z.-S.); epatelarou@hmu.gr (E.P.); 2 Clinic of Social and Family Medicine, School of Medicine, University of Crete, 70013 Crete, Greece; mechili@univlora.edu.al; 3 Department of Healthcare, Faculty of Public Health, University of Vlora, 9401 Vlora, Albania; 4 Center for Health Services Management and Evaluation, Faculty of Nursing, National and Kapodistrian University of Athens, 15772 Athens, Greece; pegalan@nurs.uoa.gr

**Keywords:** COVID-19, attitude, compliance, knowledge, nursing students, validation

## Abstract

Background: During the COVID-19 pandemic, nursing students have had a key role in supporting the healthcare sector. They can join healthcare professionals in clinical practice or provide information to increase citizens’ levels of knowledge and their compliance with the restriction measures. The study aimed to develop and validate a tool to measure knowledge of and attitudes toward COVID-19 among nursing students in Greece. Methods: A questionnaire was developed through theoretical research and expert consultation. A cross-sectional study was conducted among 348 undergraduate nursing students of the Department of Nursing, Hellenic Mediterranean University, recruited by convenient sampling. Validity and reliability were analyzed. Results: The Kaiser–Meyer–Olkin measure was 0.84, indicating that the sample size was adequate for factor analysis. In addition, the p-value for Bartlett’s test of sphericity was <0.001, denoting that the correlation matrix was suitable for factor analysis. The construct validity of the questionnaire was determined through exploratory factor analysis (EFA), which revealed that 16 items lead to four factors: knowledge, attitude toward restriction measures, compliance with them, and volunteering. One of the key findings of this study was that participants preferred to receive information from valid sources rather than social media during the crucial period of the “infodemic”. Conclusions: The questionnaire was shown to have satisfying psychometric properties and, therefore, can be used as a tool in future research in the area of nursing students’ knowledge, attitudes, compliance, and volunteering during the COVID-19 pandemic.

## 1. Introduction

The COVID-19 pandemic is a social phenomenon; the first cases were identified in China in December 2019 and effected intense health, social, and demographic changes. The World Health Organization (WHO), on 30 December 2019, received a media statement by the Wuhan Municipal Health Commission regarding cases of ‘viral pneumonia’ in Wuhan, People’s Republic of China, while on 30 January 2020, WHO announced a public warning regarding a health emergency of international concern [1]. Thus, on 11 February 2020, the “viral pneumonia” was named “COVID-19” and on 29 February 2020, the first considerations were published for the quarantine of individuals in the context of containment measures for the coronavirus disease [2]. By 30 September 2020, 188 countries have reported as being affected by the coronavirus, with a global number of 34 million confirmed cases, and more than one million global deaths [3]. Currently, the most affected countries worldwide are the US, India, Brazil, Russia, Colombia, Peru, and Spain [3].

The Greek government, on 10 March 2020, in response to the preventive measures against COVID-19, imposed the closure of all educational institutions and, a few days later, the suspension of arts and sports events, while from 23 March to 4 May, a confinement measure was announced accompanied by strict bans for movement [4]. Generally speaking, the Greek authorities received affirmative comments for the decision to quickly enforce restrictive measures for limiting the spread of the disease in the country [5]. In spite of the aforementioned measures, by 30 September 2020, Greece counted 18,475 confirmed cases of COVID-19 and 391 deaths [3]. Additionally, based on the local situation, restrictions were imposed in certain regions [6].

While this life-threatening pandemic rapidly spreads around the world and causes millions of deaths and observed cases, WHO highlights the threat of “another dangerous virus” called an “infodemic” [7]. This term refers to the fake news and rumors in the context of the misinformation that feeds confusion against slowing the spread of disease [7]. With respect to this phenomenon, WHO states that reducing misconceptions and confusion about the virus and dealing effectively with the vast amount of valid and invalid coronavirus information is a matter of necessity [7] because “misinformation costs lives” [8]. There is evidence showing the strong positive correlation between knowledge and attitude [9], and in the case of the pandemic, the more knowledgeable the citizens are, the more positive attitude they hold toward COVID 19-related measures and recommendations for health behavioral changes as preventive strategies [10]. Indeed, knowledge is clearly stated by researchers as the key component of evidence-based practice, not only in the area of COVID-19 but also during their training as future health professionals [11,12].

According to Nutbeam (2000), health literacy is a combination of three different domains (functional, interactive, and critical) [13]. Having a poor literacy level leads to belief in myths, unreliable information, and fiction over facts. This behavior does not impact just the believers of these stories but also their close environment and entire society [14]. During the COVID-19 epidemic, several studies have focused on health literacy. However, it still remains an underestimated public health issue [10]. In a study conducted in Vietnam, medical students were less frightened due to health literacy [15]. Another study concluded that people with poor health literacy were likely to be more confused about COVID-19 information [8]. It is clear that an increase in the general population’s knowledge and health literacy is of paramount significance for managing the epidemic and for controlling and preventing its spread [16].

Health professionals, education providers, and health science students have a key role in increasing the citizens’ level of knowledge, the implementation of the pandemic measures, and compliance with them [17]. Due to the lack of healthcare personnel, in many countries, final-year medical and nursing students were invited to voluntarily join the frontline healthcare workforce in the COVID-19 battle, in order to enhance health sectors during this public health crisis. In any case, researchers claim that, even if medical students are not involved in clinical practice during the COVID-19 outbreak, they play a key role in serving as information providers [17]. Therefore, it is of major importance to avoid misconceptions and myths, identify students’ possible knowledge gaps, encourage them as future health providers to search, critically appraise them, and adopt the new evidence in order to make informed decisions [8,18].

To our knowledge, no fully validated tool exploring nursing students’ knowledge of and attitudes toward the COVID-19 public health crisis exists. Due to a shortage of appropriate research tools for answering this research question, we aimed to develop and validate a new instrument for the purpose of this study.

## 2. Material and Methods

### 2.1. Development of Survey Questionnaire

The study questionnaire, which comprised 24 items, was constructed using reference materials, guidance, and information on COVID-19 developed by WHO, the CDC, and National Health Services (NHS). The survey covered the domains of student demographics, general awareness, information sources, knowledge, and attitude toward COVID-19, as well as level of adherence to the restriction measures. The tool was constructed with input by diverse public health professionals and professors. The first version of the questionnaire was validated by face and content validation methods by five selected experts (two nurses, one physician, and two faculty members). This was done in order to assess its readability and validity before pilot testing among ten randomly selected nursing students to confirm the clarity and acceptability [19]. Finally, all the participants’ comments were incorporated and led to a new modified version of the tool with a better understanding and a more suitable order of questions. The final survey link was delivered to students via the educational platform of the institute.

### 2.2. Validation Process—Pilot Study

The questionnaire was evaluated for face validity by an interdisciplinary team of ten senior researchers (experts) in the fields of education and community nursing. Special attention was given to item construction, in order to avoid ambiguity or incomprehensibility [20]. The experts involved in the process critically scrutinized the scales on completeness and the items in terms of possible misunderstandings or ambiguities.

A pilot study with 30 students was conducted in order to estimate the face validity of the questionnaire. Students were asked to participate in the pilot study by completing an online Google Form developed for the purposes of the present study. Special attention was given to item construction to avoid ambiguity or incomprehensibility [20]. The participants carefully checked the scales on completeness and the items in terms of possible misunderstandings or ambiguities. All the items were clear and comprehensive, and finally, only a few syntax corrections were made by the researchers. All returned questionnaires had all items answered and were used for the statistical analysis.

### 2.3. Full Implementation Study

Following the validation process, a cross-sectional study was conducted in Greece during the period of total lockdown due to the pandemic (April–May 2020). Data were collected from April 27 to May 5 by using an online survey, from a convenience sample of undergraduate students who were attending distance classes organized by the university (Hellenic Mediterranean University). Participants were informed about all aspects of the study and voluntarily confirmed their willingness to participate. No personal data were recorded and all questionnaires were completed anonymously. Study approval was obtained by the Hellenic Mediterranean University ethical committee (ethical number 16/27.04.2020).

### 2.4. Statistical Analysis

The construct validity of the questionnaire was estimated with exploratory factor analysis, identifying the underlying factors. The varimax rotation method was used to identify correlations between items and construct the factors. Accordingly, the level for acceptable factor loading was set at >0.4 and for acceptable eigenvalues, set at >1. The Kaiser–Meyer–Olkin test was also used to measure the adequacy of the sample size for factor analysis, with values >0.7 considered as acceptable. Bartlett’s test of sphericity was applied to estimate the covariance between the items and values <0.05 indicated that the correlation matrix was suitable for factor analysis. Internal consistency for the factors was measured with the use of the raw coefficient alpha and values >0.7 were considered as acceptable.

For each factor that emerged from factor analysis, a total score was calculated by adding the answers in the factor’s items and dividing by the number of items. Thus, a total score from 1 to 5 was created, with higher values indicating greater agreement.

Continuous variables are presented as mean (standard deviation), while categorical variables are presented as numbers (percentages). The Kolmogorov–Smirnov test (*p* > 0.05) was used to test the normality assumption for the continuous variables. Bivariate analyses between demographic characteristics and total factor scores included a Student’s *t*-test, Spearman’s correlation coefficient, and Pearson’s correlation coefficient. The Student’s *t*-test was used to compare a continuous variable with a dichotomous one, while Spearman’s correlation coefficient was used to correlate a continuous variable with an ordinal one. Furthermore, the correlation between two continuous variables that followed normal distribution was assessed with Pearson’s correlation coefficient. Then, multivariable linear regression was performed with total factor scores as the dependent variables. Accordingly, the backward stepwise linear regression was applied and the coefficients’ beta, 95% confidence intervals, and *p*-values were calculated. All tests of statistical significance were two-tailed, and *p*-values < 0.05 were considered as statistically significant. Statistical analysis was performed with the IBM SPSS 21.0 (IBM Corp. Released 2012. IBM SPSS Statistics for Windows, Version 21.0 Armonk, NY, USA).

## 3. Results

### 3.1. Demographic Characteristics

From the 451 students initially approached, 348 of them completed the questionnaire (a response rate of 77.16%) and their demographic characteristics are presented in Table 1. The mean age was 23.6 years, while the majority of students were female (84.8%), single (91.7%), and living with others during pandemic (92%) in Crete (65.5%). 55.2% lived with high-risk groups, 29.9% worked before the pandemic, and 10.9% have been working during the pandemic.

### 3.2. Factor Analysis

The Kaiser–Meyer–Olkin measure was 0.84, indicating that the sample size was adequate for factor analysis. Additionally, the p-value for Bartlett’s test of sphericity was <0.001, denoting that the correlation matrix was suitable for factor analysis. The results of the exploratory factor analysis are presented in Table 2. There were four factors, including the 16 items out of the 24 questionnaire items. This four-factor model explained 50% of the questionnaire’s variance. According to common sense and the meaning of items, we characterized the factors as the following: (a) COVID-19 knowledge, (b) Attitudes toward restriction measures, (c) Compliance with restriction measures, (d) Volunteering.

### 3.3. Reliability Analysis

The reliability analysis for the questionnaire is presented in Table 3. According to the raw coefficient alpha and the Spearman–Brown coefficient, the questionnaire developed very good reliability. In particular, all raw coefficients alpha values and Spearman–Brown coefficients were >0.70, except one. The raw coefficient alpha for the overall instrument was 0.80 and the Spearman–Brown coefficient was 0.77. The raw coefficient alpha values for the four factors that emerged from the factor analysis ranged from 0.71 to 0.78.

### 3.4. Descriptive Statistics

Descriptive statistics for the 24 questionnaire items and the four factors are presented in Table 4. Mean total scores for the four factors were above the mid-point value (= 3) of the scale, indicating high knowledge levels (mean = 4.22), positive attitudes toward restriction measures (mean = 4.14), high levels of compliance with restriction measures (mean = 4.12), and intentions of students to volunteer in clinical settings (*n* = 3.34). Regarding other aspects of the questionnaire, students preferred to be more informed by official organizations (e.g., WHO, Centers for Disease Control and Prevention, etc.) (mean = 3.87), public media (mean = 3.35), and electronic databases (e.g., PubMed) (mean = 3.21), compared with social media (e.g., Facebook, Instagram, etc.) (mean = 2.46). Moreover, students believed that quarantine measures could have a moderate effect on their health (mean = 2.93), while the COVID-19 pandemic could change people’s lives in a significant way (mean = 3.90).

### 3.5. Bivariate and Multivariable Analysis

Bivariate analyses between demographic characteristics and the factors’ scores that emerged from factor analysis are shown in Table 5. There were no significant associations between demographic characteristics and COVID-19 knowledge. Students that lived with others during the pandemic had more positive attitudes toward the restriction measures (*p* = 0.04), while females showed greater compliance with restriction measures (*p* = 0.02). In bivariate analyses, age, marital status, maternal educational level, and working before the pandemic were associated with volunteering. However, in the multivariable linear regression analysis, only increased age was associated with increased intention to volunteer (coefficient beta = 0.02, 95% confidence interval = 0.004 to 0.03, *p* = 0.011).

## 4. Discussion

The abrupt spread of the COVID-19 pandemic worldwide and the declaration of the disease as a Public Health Emergency of International Concern were bad omens for public health. Healthcare providers and health science students were always at high risk of infectious diseases and, conclusively, their knowledge of and their attitudes toward the new virus are of major importance for future interventions and health policy planning.

According to our knowledge, this is the first study to develop a tool with these dimensions investigating Greek nursing students’ views during the COVID-19 confinement. In this study, which was conducted one month after the announcement of lockdown in Greece, the researchers attempted to investigate COVID-19-related knowledge, the attitude toward this new situation, the level of adherence towards the restriction measures, and volunteerism among nursing students. The developed instrument was comprehensively tested and showed satisfactory psychometrical properties, and it can be used as a valid research tool in future studies in this field. The instrument accounted for 50% of the total variance, indicating that the four factors model was statistically appropriate, including COVID-19 knowledge, attitudes toward restriction measures, compliance with restriction measures, and volunteering. Regarding the dimension of COVID-19 knowledge, we conclude that knowledge is an essential issue, as has been confirmed by several studies [21,22,23,24]. Increased awareness and promotion of positive attitudes among students are imperative to changing students’ health practices and improving compliance with preventive measures. Positive attitudes and compliance with restriction measures could significantly improve the public’s preventive health behaviors and the preparedness for COVID-19 [21,22,23,25,26]. Finally, a relatively new issue emerged from our factor analysis: the factor “volunteering”. Health care students internationally volunteered to assist in hospitals due to the COVID-19 pandemic, providing crucial aid to hospital functioning and patients’ care in healthcare systems [27,28].

One of the main results of this study was the students’ high knowledge levels (mean = 4.22), as well as their preference to get informed by official organizations and official electronic databases compared with social media. Recent studies show the importance of the need for health science students to be well-informed regarding the symptoms of COVID-19 and prevention strategies [17]. Further, a study in Turkey regarding nursing students revealed that almost half of the respondents (48%) were well-informed about the coronavirus disease, despite the fact that knowledge was associated with an increase in their stress levels [29]. Furthermore, a study based on medical and health students in India found a lack of availability of credible knowledge, with the majority (65.17%) getting informed through social media, and a small percentage of them (11.47%) not informed regarding the pandemic [19]. Notably, there is evidence indicating that health science students have higher levels of knowledge compared with social science students [30], and also have an obligation to adopt true knowledge and disseminate valid evidence regarding the spread of the virus [17].

Literature highlights the need for students’ attention to the value of the knowledge refinement process and evidence-based answers through critical appraisal of the information before applying or sharing it [31]. At the same time, authors in cooperation with public health organizations and WHO suggest frameworks, strategic partnerships, and coordinated actions for infodemic management, involving health professionals, students, researchers, and stakeholders [32,33,34]. As a further step, researchers claim that virus-related knowledge and health literacy are needed in order to achieve higher levels of compliance with restriction measures to control citizens’ fear, resist the infodemic, and to promote citizens’ trust in reliable information and recommendations [15].

Moreover, in this study, most of the participants hold a positive attitude toward restriction measures. Similarly, a national study in the United Kingdom conducted among final year medical students showed that the majority (93.6%) believed that the measures during the pandemic are necessary [35]. In contrast, in the US, 37.8% of the students presented unwillingness to comply with restriction measures, while in Cyprus, researchers showed that women and individuals over the age of 30 were more likely to implement the measures [36,37].

Additionally, the present study reveals the intentions of students to volunteer in clinical settings. This is in line with previous studies’ findings in Spain, which found that a high percentage of students expressed the desire to assist nurses in providing patient care during the pandemic [38]. Internationally, nursing and medical students were willing to volunteer for certain tasks in order to assist the healthcare system and support the COVID-19 response effort [39]. Volunteerism in the period of the COVID-19 crisis, although can cause uncertainty and fear for students acting in the frontlines, is still considered a valuable lesson for their future interprofessional practice, and therefore, a high percentage of students report a willingness to get involved [28,35].

Moreover, students reported that the COVID-19 pandemic could change people’s lives in a significant way, which is in line with previous findings. Similarly, a study conducted in Hong Kong showed that the vast majority of participants (97%) were worried about COVID-19 and its impact on their daily routines [40], while another study conducted in the United Kingdom reported medical students’ disappointment due to the worldwide travel restriction [35]. 

In the present study, a portion of the participants alleged that the quarantine measures could have a moderate effect on their health (mean = 2.46). Rawls and Gibson stated that the pandemic may cause negative economic, psychological, and cultural consequences [41], as this situation affects students’ psychological status and creates anxiety and fears for their future careers [38,42]. In addition, there is evidence linking social isolation with uncertainty, insecurity, and instability to students, as well as with emotional difficulties during student life [36]. Generally speaking, studies have shown that the COVID-19 pandemic and measures to control it have a great impact on individuals’ quality of life and mental health, while in a study conducted in the general population of Cyprus, the psychological morbidity was associated with being a university student [42].

The present study has some strengths, as well as limitations. A possible limitation of our study is its cross-sectional study design, which does not allow us to assess possible changes in nursing students’ attitudes, their levels of knowledge, or their adherence to restrictions over the different stages of the COVID-19 pandemic. On the other hand, the cross-sectional design allows researchers to use real-time data and clearly depicts the participants’ knowledge and attitudes at that point in time, during the confinement. It is worth noting that to our knowledge, this is the first study to develop and validate a tool to assess nursing students’ knowledge and attitudes toward COVID-19 in a university environment during the period of quarantine. Another limitation is that we did not perform a confirmatory factor analysis since we did not have a prior theory regarding the number and the structure of the factors. Thus, there is a need for further studies in this field to confirm and expand our findings.

## 5. Conclusions

The questionnaire developed proved to have satisfactory psychometric properties in terms of validity and reliability. This instrument can be used as a tool in future research among different student populations in the area of knowledge, attitudes, compliance, and volunteering during the COVID-19 pandemic.

## Figures and Tables

**Table 1:** Students’ demographic characteristics.

Items	*N*	%
Sex		
Male	53	15.2
Female	295	84.8
Age ^a^	23.6	7.4
Marital status		
Single	319	91.7
Married	24	6.9
Divorced	5	1.4
Paternal educational level		
Basic education	113	32.5
High school	139	39.9
University degree	76	21.8
M.Sc./Ph.D. degree	20	5.7
Maternal educational level		
Basic education	63	18.1
High school	169	48.6
University degree	105	30.2
M.Sc./Ph.D. degree	11	3.2
City of residence during pandemic		
Crete	228	65.5
Athens	55	15.8
Other	65	18.7
Living status		
Alone	28	8.0
With others	320	92.0
Living with people at high-risk groups		
Yes	192	55.2
No	156	44.8
Working status before the pandemic		
Yes	104	29.9
No	244	70.1
Working status during the pandemic		
Yes	38	10.9
No	310	89.1

^a^ mean, standard deviation.

**Table 2:** Exploratory factor analysis for the 24 questionnaire items.

Items	Factors Derived from the Exploratory Factor Analysis			
	1	2	3	4
	Knowledge	Attitudes	Compliance	Volunteering
I am aware of COVID-19 infection symptoms.	0.73			
I am aware of the factors affecting COVID-19 transmission.	0.69			
I am aware of the correct use of protective equipment in cases of the epidemic.	0.65			
I know what to do if I come in contact with a confirmed case.	0.74			
I know which groups are at high risk for serious disease from COVID-19.	0.64			
I know where to search for updated evidence regarding the COVID-19 epidemic.	0.56			
Compliance with self-protective/restriction measures is of high importance for limiting the spread.		0.57		
My country announced the restriction measures in a timely manner.		0.79		
The measures that have been implemented in my country against COVID-19 make me feel safe.		0.74		
Strict compliance with restriction measures is imperative for securing public health.		0.69		
I personally strictly adopt the restriction measures for social isolation, and I remain at home.			0.74	
When I am outside my house, I keep safe distances.			0.71	
I perform hand hygiene according to the guidelines in my daily life.			0.68	
I feel able to volunteer my services in clinical practice.				0.82
I would like to volunteer my services in clinical practice for the treatment of the COVID-19 epidemic.				0.82
I am afraid to offer my services voluntarily in clinical practice for the treatment of the COVID-19 epidemic (reversal).				0.74
My main source of information is social media (Facebook, Instagram, etc.).				
I get informed through official organizations (National Public Health Organization, World Health Organization, CDC, etc.).				
I search for reliable information about COVID-19 in scientific articles from bibliographic databases (e.g., PubMed).				
I get informed about COVID-19 mainly through the media.				
Social distancing (quarantine) can damage my health (reversal).				
I feel able to appropriately adopt hygiene protection measures and equipment (e.g., mask, gloves).				
Guidelines regarding hygiene rules and restriction measures are clear and there is no confusion among citizens.				
I believe that this epidemic will significantly change our way of life from now on.				

Values express loadings.

**Table 3:** Reliability analysis for the questionnaire.

Items	Factors Derived from the Exploratory Factor Analysis				Overall Instrument
	1	2	3	4	
	Knowledge	Attitudes	Compliance	Volunteering	
Raw coefficient alpha	0.78	0.71	0.73	0.78	0.80
Spearman–Brown coefficient	0.73	0.73	0.70	0.71	0.77
Part 1	0.73	0.41	0.72	0.81	0.70
Part 2	0.70	0.72	1	1	0.73

**Table 4:** Descriptive statistics for the 24 questionnaire items and the four factors.

Items	Mean	SD	Median	Min	Max
I am aware of COVID-19 infection symptoms.	4.27	0.6	4	3	5
I am aware of the factors affecting COVID-19 transmission.	4.27	0.7	4	2	5
I am aware of the correct use of protective equipment in cases of the epidemic.	4.25	0.7	4	2	5
I know what to do if I come in contact with a confirmed case.	4.14	0.8	4	1	5
I know which groups are at high risk for serious disease from COVID-19.	4.40	0.6	4	2	5
I know where to search for updated evidence regarding the COVID-19 epidemic.	3.97	0.8	4	1	5
Compliance with self-protective/restriction measures is of high importance for limiting the spread.	4.22	0.9	4	1	5
My country announced the restriction measures in a timely manner.	4.33	0.8	4	1	5
The measures that have been implemented in my country against COVID-19 make me feel safe.	3.74	0.9	4	1	5
Strict compliance with restriction measures is imperative for securing public health.	4.26	0.8	4	1	5
I personally strictly adopt the restriction measures for social isolation and remain at home.	3.97	0.9	4	1	5
When I am outside my house, I keep safe distances.	4.08	0.8	4	1	5
I perform hand hygiene according to the guidelines in my daily life.	4.32	0.8	4	1	5
I feel able to volunteer my services in clinical practice.	3.48	1.1	3	1	5
I would like to volunteer my services in clinical practice for the treatment of the COVID-19 epidemic.	3.34	1.2	3	1	5
I am afraid to offer my services voluntarily in clinical practice for the treatment of the COVID-19 epidemic (reversal).	2.78	1.2	3	1	5
My main source of information is social media (Facebook, Instagram, etc.).	2.46	1.2	2	1	5
I get informed through official organizations (National Public Health Organization, World Health Organization, CDC, etc.).	3.87	0.9	4	1	5
I search for reliable information about COVID-19 in scientific articles from bibliographic databases (e.g., PubMed).	3.21	1.1	3	1	5
I get informed about COVID-19 mainly through the media.	3.35	1.1	4	1	5
Social distancing (quarantine) can damage my health (reversal).	2.93	1.1	3	1	5
I feel able to appropriately adopt hygiene protection measures and equipment (e.g., mask, gloves).	4.40	0.7	4	1	5
Guidelines regarding hygiene rules and restriction measures are clear and there is no confusion among citizens.	3.27	1.0	3	1	5
I believe that this epidemic will significantly change our way of life from now on.	3.90	1.0	4	1	5
COVID-19 knowledge	4.22	0.2	4.2	3	5
Attitudes toward the restriction measures	4.14	0.6	4.3	1.3	5
Compliance with restriction measures	4.12	0.7	4	1	5
Volunteering	3.34	1.0	3.3	1	5

SD: standard deviation.

**Table 5:** Bivariate analysis between demographic characteristics and factors’ scores that emerged from factor analysis.

Items	Knowledge			Attitudes			Compliance			Volunteering		
	Mean	SD	*p*	Mean	SD	*p*	Mean	SD	*p*	Mean	SD	*p*
Sex			0.5 ^a^			0.5 ^a^			**0.02 ^a^**			0.8 ^a^
Male	4.2	0.5		4.1	0.6		3.9	0.7		3.4	0.9	
Female	4.2	0.5		4.1	0.6		4.2	0.7		3.3	0.9	
Age		0.04 ^b^	0.5 ^b^		0.03 ^b^	0.5 ^b^		0.05 ^b^	0.3 ^b^		0.14^b^	**0.01 ^b^**
Marital status			0.9 ^a^			0.7 ^a^			0.7 ^a^			**0.03 ^a^**
Single/divorced	4.2	0.5		4.1	0.6		4.1	0.7		3.3	0.9	
Married	4.2	0.5		4.1	0.8		4.2	0.5		3.8	0.8	
Paternal educational level		0.02 ^c^	0.6 ^c^		0.04 ^c^	0.5 ^c^		0.06 ^c^	0.3 ^c^		−0.07 ^c^	0.18 ^c^
Maternal educational level		−0.11 ^c^	0.2 ^c^		0.01 ^c^	0.8 ^c^		−0.02 ^c^	0.7 ^c^		−0.14 ^c^	**0.01 ^c^**
Living status			0.5 ^a^			**0.04 ^a^**			0.9 ^a^			0.8 ^a^
Alone	4.2	0.5		3.9	0.9		4.1	0.6		3.4	0.9	
With others	4.2	0.5		4.2	0.6		4.1	0.7		3.3	0.9	
Living with people in high-risk groups			0.9 ^a^			0.2 ^a^			0.3 ^a^			0.7 ^a^
Yes	4.2	0.5		4.1	0.6		4.1	0.7		3.3	0.9	
No	4.2	0.5		4.2	0.7		4.2	0.6		3.4	0.9	
Working status before the pandemic			0.2 ^a^			0.2 ^a^			0.7 ^a^			**0.02 ^a^**
Yes	4.3	0.5		4.1	0.8		4.1	0.6		3.5	0.9	
No	4.2	0.5		4.2	0.6		4.1	0.6		3.3	0.9	
Working status during the pandemic			0.3 ^a^			0.8 ^a^			0.6 ^a^			0.1 ^a^
Yes	4.3	0.5		4.1	0.8		4.1	0.5		3.6	0.9	
No	4.2	0.5		4.1	0.6		4.1	0.5		3.3	0.9	

^a^ Student’s t-test, ^b^ Pearson’s correlation coefficient, ^c^ Spearman’s correlation coefficient, SD: standard deviation, *p*: *p*-value, Bold: *p*-values < 0.05.
